# Factors affecting implementation of tuberculosis contact investigation and tuberculosis preventive therapy among children in Sabah, East Malaysia: A qualitative study

**DOI:** 10.1371/journal.pone.0285534

**Published:** 2023-05-11

**Authors:** Michelle May D. Goroh, Christel H. A. van den Boogaard, Khamisah Awang Lukman, Christopher Lowbridge, Wong Ke Juin, Timothy William, Mohammad Saffree Jeffree, Anna P. Ralph

**Affiliations:** 1 Department of Public Health Medicine, Faculty of Medicine & Health Sciences, Universiti Malaysia Sabah, Kota Kinabalu, Sabah, Malaysia; 2 Menzies School of Health Research, Charles Darwin University, Darwin, Australia; 3 Sabah Women and Children’s Hospital, Ministry of Health, Kota Kinabalu, Sabah, Malaysia; 4 Gleneagles Hospital Kota Kinabalu, Kota Kinabalu, Sabah, Malaysia; Stellenbosch University, SOUTH AFRICA

## Abstract

Contact investigation and TB preventive treatment of children under five years of age who are close contacts of a TB case is a key component of TB prevention. However, the uptake of TB preventive treatment is low in many high-TB burden settings. This study explores factors affecting the implementation of TB contact investigation and preventive treatment among children in Malaysia’s city of Kota Kinabalu, Sabah State. This study was conducted in three primary health clinics between 2019 and 2020. We purposively sampled 34 parents and guardians of child contacts eligible for TB preventive treatment, and 25 healthcare providers involved in the management of child contacts. We conducted thematic analysis of semi-structured interviews and focus group discussions to illicit factors affecting implementation and uptake of TB contact investigation and TB preventive therapy. Six main themes emerged from the analyses–four of these relating to contact investigation and two relating to TB preventive therapy. Factors affecting TB contact investigation were addressed under system related factors (external factors, stakeholder collaboration, healthcare workers’ and clients’ concerns), clinic related factors (perceived performance, clinic schedule, and space), healthcare worker related factors (cooperation, commitment, knowledge, misconception, counselling and communication) and patient and contact related factors (cooperation and commitment). Factors affecting TB preventive treatment delivery were addressed under guardian related factors (cooperation, commitment, knowledge and misconception) and treatment related factors (child-friendly form and adverse effects). To address gaps and barriers identified in our study, we recommend developing system capacity to maintain routine contact investigation and preventive treatment in the context of external program risks, providing training to healthcare workers to address misconceptions, safeguarding vulnerable clients against the risk of detention and deportation while accessing care, ensuring public and private services are provided regardless of migration status, and improving processes and resources for contact investigation and preventive treatment.

## Introduction

Tuberculosis (TB) is a preventable and curable disease, yet it is estimated that 1.5 million people die from TB each year [1]. Close contacts of infectious TB cases are known to be at high-risk of TB infection and progression to TB disease [2]. Contact investigation, including screening for TB disease and TB infection among close contacts of people with infectious TB is a key component of TB prevention. Contact investigation enables early identification of TB disease, thus decreasing its severity and reducing the risk of transmission to others, and identification of latent TB infection (LTBI) which facilitates the provision of TB preventive treatment [3]. Children less than five years of age are at increased risk of severe forms of TB, such as meningitis and disseminated TB [4]. The World Health Organization (WHO) strongly recommends that all children aged less than five years who are household contacts of a person with bacteriologically confirmed pulmonary TB are offered TB preventive treatment [5, 6].

TB is a disease of public health significance in Malaysia, with 25 600 cases recorded nationally in 2019, and an estimated incidence rate of 92 cases per 100 000 giving it a status of intermediate TB burden country. While Malaysia is successfully reducing TB related mortality, TB incidence is not decreasing in line with global End TB Strategy milestones [3]. The state of Sabah, located in East Malaysia, has historically had a higher burden of TB. While Sabah State accounts for only 10% of the country’s total population, in 2018, the case notification rate in Sabah was 47% higher than that of the national case notification rate of Malaysia [7].

While TB rates in other parts of the country have fallen over the past decade, high TB case notification rates have been sustained in Sabah, between 2012 and 2018 there were 33 193 cases of TB reported (128 cases per 100 000 population). Most of these cases (89%) were detected passively [8]. Case detection among children was very low, with children aged less than 15 years accounting for only 4.6% of cases, despite that age-group accounting for 24% of the Sabah population and global estimates of disease burden suggesting that this age-group accounts for 11% of all TB cases [9].

**Fig 1:**
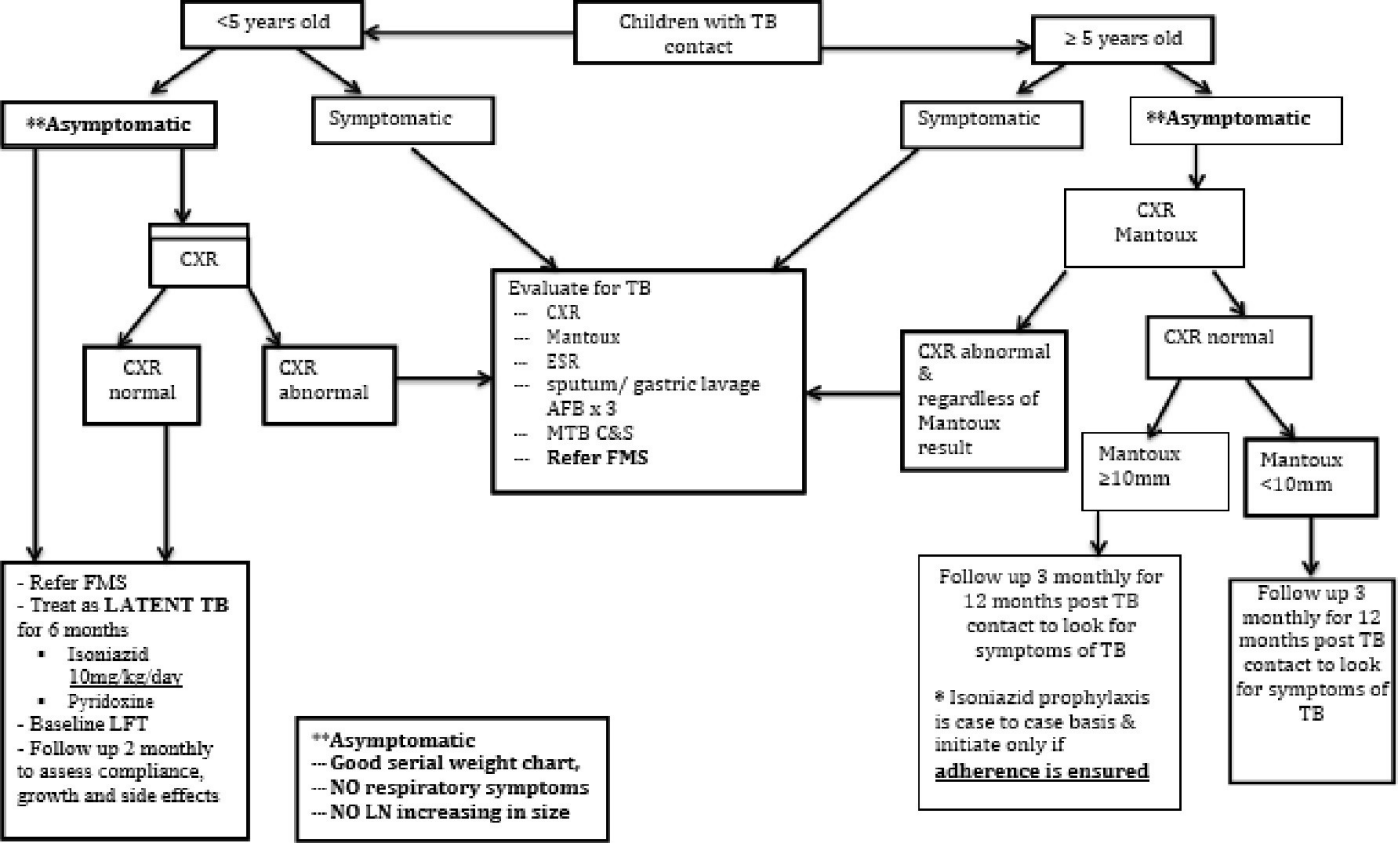
Latent TB management flowchart in Sabah.

To identify contacts, a list with confirmed TB patients diagnosed at the TB clinic is forwarded to health inspectors working for the government. Then, these health inspectors perform contact tracing by visiting the homes or workplace of TB patients to list the patient’s contacts and details. During this visit, the identified contacts receive a notification form to attend the TB clinic for investigation within 2 weeks of notification. Tuberculosis Preventive Therapy (TPT) is offered to child contacts less than 5 years of age who are household or close contacts of people with TB and who are not symptomatic and have been confirmed not to have active TB disease after going through a series of investigations and assessment by a Family Medicine Specialist (FMS). Children who receive TPT should be followed up at least once every two months until treatment is completed. The flowchart for latent TB infection management in Sabah is shown in Fig 1.

Despite recommendations to provide TPT to eligible children aged less than five years who are in close contact with an infectious TB case, TPT delivery in high-burden settings remains poor, whereby only 21% of children eligible for TPT had documentation of TPT delivery [10] as opposed to the recommended target of 90% and more by WHO [4]. A previous study to identify factors affecting continued participation in TB contact investigation in Kota Kinabalu, Sabah revealed that the contacts who were notified to attend the TB clinic directly by a health inspector or who had a close relative diagnosed with TB were more likely to attend the clinic for TB investigation [11].

There is a need to improve the contact investigation and TB preventive treatment cascade of care for child contacts of people with TB in Sabah, given the low uptake of TB preventive treatment. Our previous work has identified local issues and barriers to accessing medical care in Sabah, [11–13] and there is a need to better understand the barriers and challenges to implementing policy into practice in this setting. This qualitative study aimed to explore the factors affecting the implementation of TB contact investigation and TPT among child contacts in Kota Kinabalu, Sabah.

## Materials and methods

### Study design

This is a qualitative study which is part of an implementation research using hybrid type II design, to improve quality of contact management and implementation of TPT among child TB contacts in Kota Kinabalu, Sabah. Grounded theory methodological approach was applied to explain what were the facilitators and barriers to implementation of TPT among contacts and also healthcare providers.

### Study setting

This study was conducted from January 2019 to December 2020 in three primary healthcare clinics in Kota Kinabalu, Sabah (for purposes of anonymity coded as facility A, facility B and facility C). These facilities were purposively selected based on physical location, size and the high volume of TB cases and contacts reported during recent years.

### Participants and data collection

This study included 25 healthcare workers involved in the management of TB in the three participating clinics (medical officers, medical assistants, health inspectors, staff nurses and community nurses) and 34 parents or guardians of TPT eligible child TB contacts who are either TB cases or contacts themselves selected via purposive sampling.

Data were gathered through semi-structured interviews with healthcare workers and parents or guardians of child TB contacts. Interviews were conducted face-to-face and recorded at the selected clinics by trained research nurses fluent in both English and the local language, Bahasa Malaysia. Question and answers were translated from English to Bahasa Malaysia and vice versa by the first author (MG) with the help of two research nurses. Questions in the interview guide were open-ended, and emerging themes and hypotheses from earlier interviews were explored in subsequent interviews. All participants were included in the two Focus group discussions. FGDs and feedback sessions were conducted among healthcare workers in each clinic during regular site visits for further exploration and validation of information. Discussions were moderated by the first author and assisted by research nurses. Notes on informal conversations and observations were also taken.

### Analysis

Qualitative data was transcribed by study nurses, translated into English prior to analysis, and organised in NVivo® qualitative data analysis Software for Windows; QSR International Pty Ltd. Version 12 Plus. A grounded theory methodological approach was applied to explain the factors affecting the implementation of contact investigation and TPT among healthcare providers and guardians by presenting a conceptual framework. Coding consistency was assessed by comparing two rounds of coding carried out by two independent coders (MG and CB). Coding of transcripts from interviews and focus group discussions was done to condense the data into identifiable topics and allow identification of patterns and outlying findings. The data collection, coding and categorizing process was continued until data saturation was obtained. The final stage involved synthesizing the findings and identifying emerging themes to create a clearer understanding of the barriers and facilitators at each level of the TB control program.

### Ethics

This study was registered with the Malaysian National Medical Research Registry (NMRR) and has obtained approval from the Ministry of Health Medical Research Ethics Committee (NMRR ID: NMRR-17-2139-37893), the Human Research Ethics Committee of the Northern Territory Department of Health and Menzies School of Health Research (HREC: 2017–2960) and the Ethical Committee of Medical Research University Malaysia Sabah (JKetika 3/19 (17)). Informed oral and written consent was sought from all participants. Data confidentiality was ensured by using numeric codes to identify participants.

## Results

A total of 25 healthcare providers were involved in the study, including 17 from three participating clinics with designations ranging from medical officers, medical assistants, and nurses, as well eight health inspectors who are involved in TB contact investigation in the community. There were a total of 34 parents and guardians of children eligible for TPT participated in interviews. Characteristics of participants are displayed in Tables 1 and 2.

**Table 1:** Characteristics of parents and guardians interviewed.

Characteristics (n = 34)	Frequency (%)
**Age group**	
<20	1 (2.9)
21–30	15 (44.1)
31–40	15 (44.1)
41–50	3 (8.9)
**Gender**	
Male	8 (23.5)
Female	26 (76.5)
**Nationality**	
Malaysian	16 (47.1)
Non-Malaysian	18 (52.9)
**Case status**	
TB Patient	15 (44.1)
TB Contact	19 (55.9)
**Relationship to child eligible for TPT**	
Parent	31 (91.1)
Guardian	3 (8.9)

**Table 2:** Characteristics of healthcare workers interviewed.

Characteristics (n = 25)	Frequency (%)
**Age group**	
21–30	4 (16.0)
31–40	7 (28.0)
41–50	6 (24.0)
>50	8 (32.0)
**Gender**	
Male	9 (36.0)
Female	16 (64.0)
**Designation**	
Medical officer (Doctor)	4 (16.0)
Health inspector	8 (32.0)
Head nurse	1 (4.0)
Medical assistant	2(8.0)
Staff nurse	7 (28.0)
Community nurse	3 (12.0)
**Place of work**	
Facility A	6 (24.0)
Facility B	7 (28.0)
Facility C	4 (16.0)
District health office (Health inspectors)	8 (32.0)

### Factors affecting implementation of TB contact investigation and TPT among children

**Fig 2:**
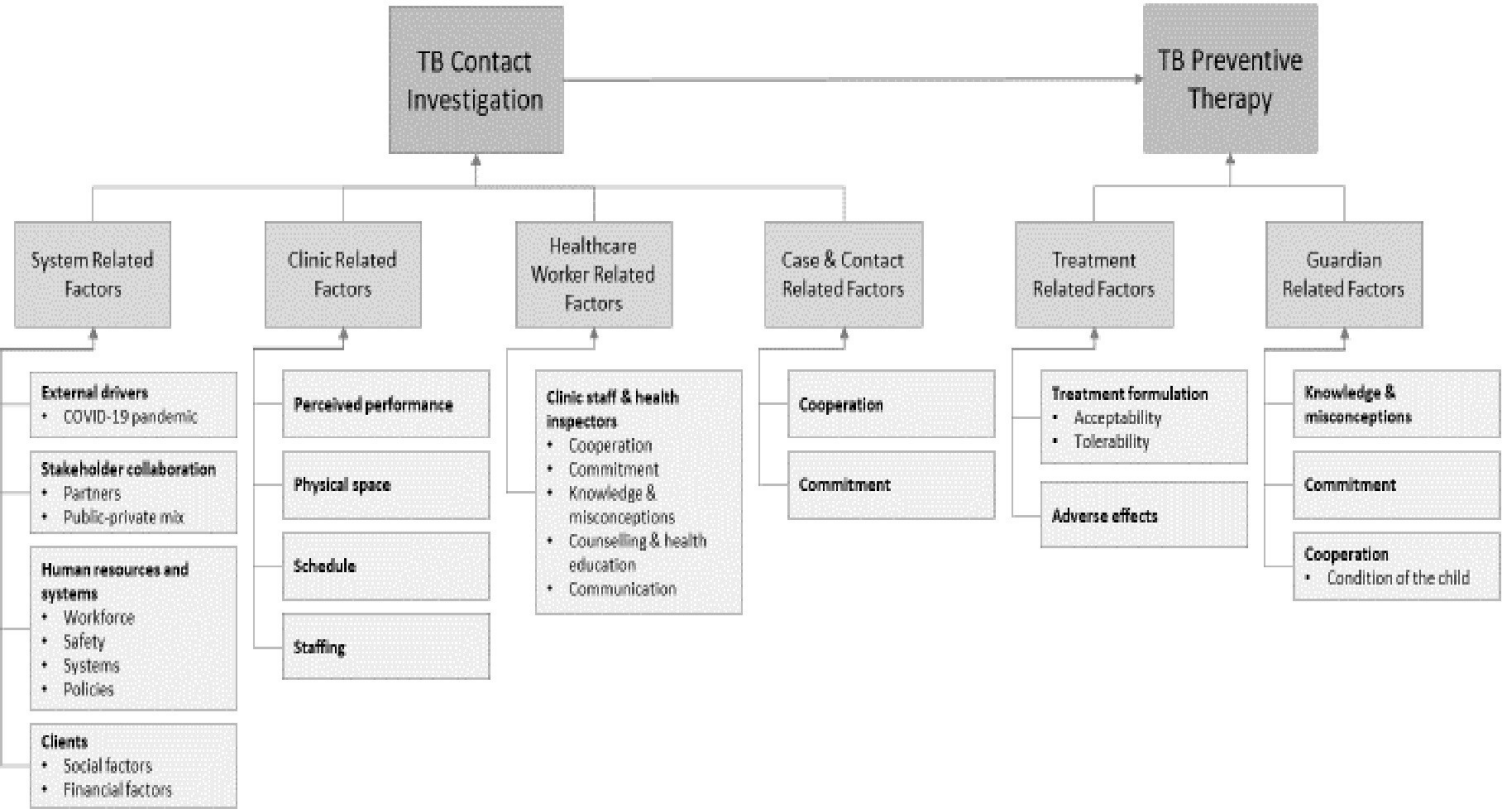
Conceptual framework of factors affecting implementation of TB contact investigation and TPT.

Six factors were identified from thematic analysis at different levels of the healthcare system. These factors are grouped and presented in the following themes: system related factors, clinic related factors, healthcare worker related factors, patient and contact related factors, guardian related factors and treatment related factors. These results are summarized and presented in a conceptual framework in Fig 2.

#### 1. System related factors

System-related factors need to be addressed and supported at the system level, which covers external factors, stakeholder collaboration, healthcare workers, and client affecting factors.

*External factors*. The external factor affecting implementation of TB contact investigation in this study was the COVID-19 pandemic. As a response to the pandemic, the government of Malaysia enforced a movement control order (MCO) as a preventive measure, which prohibited mass gatherings and restricted movement. The MCO resulted in the closure of all but essential services, which ultimately disrupted routine TB treatment and prevention services. Many healthcare staff involved in TB contact investigations were reassigned to COVID-19 response activities, and roadblocks and inter-district border closures prevented patients and contacts from attending their TB clinic appointments.

*"All is well apart from this covid-19 pandemic problem*. *This makes the number of contacts in the clinic decrease because most of the time*, *everyone is instructed to stay at home*.*" (Clinic staff)*

*"Since the COVID-19 pandemic our IK (health inspector) is always unavailable and busy*. *Also*, *due to this pandemic Covid-19*, *it’s difficult for us to do home visits*.*" (Clinic staff)*.

*Stakeholder collaboration*. Healthcare workers valued alliances with other stakeholders, such as non-governmental organizations (NGOs) and private practitioners, to provide additional support in implementing contact investigation. However, they noted the limitations of existing arrangements. In Sabah, there is an NGO formed specifically to aid the state TB control program, known as Sabah Tuberculosis Association (SABATA). This organization offers financial and welfare support to households affected by TB, but only if the patient is of Malaysian nationality possessing a legitimate identification card, or a permanent resident in Malaysia.

*"For contacts who live far from the clinic*, *they usually have transportation problem*. *We usually provide SABATA aid for them but only for the locals*.*" (Clinic staff)*

The adoption of a public-private mix system, whereby clinics without onsite x-ray facilities refer contacts to private clinics with x-ray facilities, was seen as a facilitator in contact investigation. This initiative is led by the State TB Control Unit, which identifies clinics with a high TB burden without onsite x-ray facilities, and matches them to private clinics with x-ray capacity. The State TB Control Unit subsides patients to have their x-ray at the private clinic through provision of an x-ray coupon. However, the number of participating private clinics and the number of x-ray coupons available varies year to year, based on the initiative’s fluctuations in available budget allocated by the Ministry of Health. As noted in the following section, while cooperation between sites to share facilities is valued, a preferred solution would be x-ray capability at all sites.

*"Since the government provide free coupons for a chest x-ray*, *it is feasible for them to do the x-ray in private clinics*. *The number of contacts screened is also increased*.*" (Clinic staff)*

*Healthcare workers*. Many staff identified a lack of human resources and not having diagnostic facilities onsite (Facilities B and C) as barriers to contact investigation. In contrast, staff at Facility A, the only clinic with complete diagnostic facilities onsite (x-ray and laboratory) perceived these facilities to be enablers in the contact investigation process.

*"There is a critical insufficiency of staff; [Facility B] is the clinic with the highest TB burden in KK*. *The clinic has to prioritize attending to TB cases instead of contacts which affects contact investigation*.*"* (Clinic staff)

*"In my opinion*, *the contact tracing system needs to be improved by adding the number of staff*. *We often face the problem of lack of staff to handle the contact tracing*, *so I recommend increasing the number of staff to reduce the workload*, *and contact tracing can be updated without any delay*.*"* (Health inspector)

*"One of the usual barriers faced during contact investigation is due to no x-ray in the clinic and also lack of nearby x-ray facilities*.*"* (Clinic staff)

*"Since we have x-ray and laboratory here*, *it is easy for us to implement the investigation*.*"* (Clinic staff, Facility A)

Clinic staff raised concerns about the safety and wellbeing of unregistered immigrants attending the TB clinics as a challenge to implementing contact investigation. This was because undocumented immigrants in Sabah face detention and deportation if identified.

*"Illegal immigrants are afraid that they might get caught on the way to the clinic*.*"* (Clinic staff)

*"Cases who are illegal immigrants do not have a permanent address and are always moving around as they face a high risk of being caught and getting deported if their whereabouts are known*, *thus causing the difficulty to track and trace contacts*.*" (Health inspector)*

Healthcare workers reported that not having a clear and systematic contact investigation process, with proper guidelines on TPT implementation, was a challenge to contact investigation implementation.

*"Too many work processes; first case investigation*, *then send contact notification form to the clinic*, *then input the case notification into E-notice (National Communicable Disease Control Information System*, *CDCIS) then update MyTB (National Tuberculosis Information System)*.*" (Health inspector)*

*"There are no proper guidelines or protocol to refer in terms of TPT implementation*.*" (Clinic staff*, *doctor)*

*"There’s no proper system to follow up for contact screening*.*” (Clinic staff)*

*Clients*. The role of social factors, including stigma, access to social support, and social status of undocumented non-Malaysians (illegal immigrants) in the community, was also brought up in the interviews as a contributing factor to delays in clinical evaluation and initiation of preventive therapy among TB contacts.

*“Family members have a stigma towards the case and refuse to provide any support or admit that they have been in contact*.*” (Health inspector)*

*“Case is worried of social stigma from the community and is ashamed to provide the real information for contact screening*.*” (Health inspector)*

Social support played an important role in ensuring contacts attended the TB clinic for the necessary clinic assessment and follow-up appointments.

“*Every time I need to go to the clinic*, *nobody is going to take care of my other kids so*, *I have to bring them all to the clinic*, *which is difficult for me*.*” (Parent of a child on TPT)*

*“I ask my sister-in-law to pick up the medication (TPT) on my behalf when I’m unable to collect it myself*.*” (Parent of a child on TPT)*

A lack of valid migration status of TB contacts was one of the most consistent problems identified by clinic staff. It was noted that undocumented immigrants had a social status that was deemed unfavourable by the community and often tied with stigma.

*“For non-locals*, *some do not come to the clinic for fear of being arrested by the police due to not having a valid passport*.*” (Clinic staff)*

The clients (TB patients and contacts) described financial difficulties as a barrier to attending the clinics. Cost and availability of transport to the clinic and distance from the place of residence to the clinic were reported as challenges to clinic attendance.

*“When it comes to transportation and picking up the medication*, *it is hard for me because I need to take three buses before I can go to the clinic*.*” (Parent of a child on TPT)*

*“I couldn’t go to the clinic when my husband is not around because there is no public transport there*.*” (Parent of a child on TPT)*

*“I find it hard to come to the clinic when I have no money*.*” (TB contact)*

#### 2. Clinic related factors

These factors can be influenced at the clinic level. They included the perceived performance of the clinic, the clinic schedule, and the physical space of the clinic. Clinic staff perceived deficiencies in their performance at TB contact investigation to primarily resulting from insufficient time available to carry out this activity. Clinic staff reported that this affected the mental health of some of them.

*“It’s time-consuming for contact screening and we don’t have time to update MyTB (online TB information system)*.*” (Clinic staff)*

*“The health facility is always late in filling up necessary forms and handing it to the necessary parties on time*.*” (Health inspector)*

*“A lot of the staff are stressed out and have trouble sleeping*.*” (Clinic staff)*

Clinic staff reiterated a lack of space in the TB clinics, which hindered implementation of contact investigation and their performance in delivering care to clients.

*“I have to share a clinic room with 2 other doctors and assist with other patients*. *I always avoid giving long advice to patients because it is not a priority and I’m afraid that my other colleagues and patients would get infected as our clinic room has no window*, *is air-conditioned and is very small*.*” (Clinic staff*, *doctor)*

*“It is difficult for parents or guardians to bring children for TPT as there is no parking space and the clinic is located on top of a hill despite them agreeing to start IPT for the children*.*” (Clinic staff*, *doctor)*

The healthcare workers also acknowledged issues in time management as a limiting factor in the implementation of contact tracing. A more systematic approach to scheduling clinic activity and patient appointments was seen as a potential solution to improving clinic performance.

*“The main barrier usually faced during contact investigation is the lack of time*. *Sometimes there are too many patients come in a day*. *So*, *the investigation needs to be done faster*.*”* (Clinic staff)

*“The insufficient amount of time to attend the contacts*. *Sometimes the contacts are from workplace*, *school*, *office*, *there are too many of them so we divide them into a group to come for screening*.*”* (Clinic staff)

*“No specific day for TPT follow up; we have to combine the appointments with pulmonary and extrapulmonary TB patients’ appointments which causes the parents and guardians to be unwilling as they’re afraid the children might get infected*.*”* (Clinic staff, doctor)

#### 3. Healthcare worker related factors

These factors can be influenced at the health care worker level. The barriers and facilitators which emerged in this area were: cooperation, knowledge, misconception, counselling, and communication.

*Cooperation and commitment*. Some participants perceived a lack of cooperation among parties involved and a lack of commitment to achieving the goals of TB contact investigation.

*“Investigating officers are inefficient and refuse to share and report their investigation findings with each other regularly and to supervising officer*.*” (Health inspector)*

Clinic staff, however, indicated cooperation from all parties involved in TB management to strengthen the contact investigation process.

*“So far*, *management for the contact investigation at this clinic is still at a satisfactory level*. *Each incoming contact will be screened and followed up until the end of the screening period*. *But all this requires the cooperation of many parties*, *such as patients themselves*, *close contacts*, *clinic staff*, *and health inspectors*. *It is crucial to strengthen contact tracing*.*” (Clinic staff*, *medical assistant)*

*Knowledge*. When interviewed, most healthcare workers felt they had adequate knowledge of TB transmission and disease to enable them to provide effective health education to patients and contacts. Their perception of the benefits of contact investigation was a motivating factor in their implementation of the process.

*“Tuberculosis investigation is essential to control this infectious disease*. *It is vital for us to know who lives in the same house*, *works in the same place*, *and hangs out with the patient*. *When this contact investigation is done*, *it will help reduce the risk of infection*.*” (Clinic staff*, *medical assistant)*

*“Yes*, *TB contact investigation is worthwhile*. *For their family and close contacts are able to get screened and treated earlier to prevent them for infecting other people*.*” (Clinic staff*, *staff nurse)*

However, one of the interviews revealed the potential for misconceptions to impact implementation, with one clinic staff commenting, *“If we continue to force them to take TPT and they fail to come to the clinic to get the medicine supply*, *it will bring more problems such as MDR risk*.*”*

The clinic staff also identified the inability to provide proper counselling and health education to clients as a barrier to effective contact investigation and TPT implementation.

*“It is difficult to counsel or educate non-locals*.*” (Clinic staff*, *medical assistant)*

*“On average on a day*, *I will see 20 patients with appointments and 30 walk-in patients; there is very limited time for me to give proper advice and counselling*.*” (Clinic staff*, *doctor)*

*“Commonly noted barriers include insufficient space in the clinic for counselling*.*” (Clinic staff*, *nurse)*

Healthcare workers also reported communication between relevant parties as a factor influencing contact investigation. Clinics have to send a notification form to the health inspectors in cases where contacts did not show up for their clinic appointment, and health inspectors are obliged to investigate and provide feedback to the clinic. It was revealed that frequently, the contact attended a different facility for assessment. This information was not relayed to the facility where the contact was initially registered, leading to improper documentation on contact follow-up or the inability to trace the contact.

*“Difficult to co-manage with other facilities; no communication on compliance to treatment or loss to follow up*.*” (Clinic staff*, *nurse)*

*“We try to contact them (contacts) via phone and send the TBIS-10 D form (reporting form for case or contact who can’t be reached to the district health inspector for further action*, *who will investigate and provide feedback to the clinic about the person*. *Some of the TB contacts might either have been transferred to another facility for follow up*, *go back to their hometown or are unable to be traced*.*”* (Clinic staff, medical assistant)

#### 4. Patient and contact related factors

Factors that can be influenced at the client level identified through qualitative analysis were the client’s cooperation and commitment to TB contact investigation and preventive therapy. Staff interviews reported that contacts frequently failed to attend appointments, which some interpreted as “uncooperative” behaviour.

*“A common problem in any clinic is the contact person not following instructions to come to the clinic for routine screening*.*”* (Clinic staff, medical assistant)

*“The contacts are not cooperative*. *They will always give many different excuses for not attending*.*”* (Clinic staff, nurse)

Furthermore, the cooperation of clients is also closely linked with their commitment to attend their clinic appointment and subsequent follow up.

*“Contact refused to attend screening as they “feel well” despite having explained the need for investigation*.*”* (Health inspector)

*“Contact did not attend follow up appointments*.*”* (Clinic staff)

#### 5. Guardian related factors

Guardian’s acceptability in providing preventive therapy to their children was highly influenced by their cooperation, commitment, knowledge, and misconception towards TPT.

*Cooperation and commitment*. One of the main reasons affecting the guardian’s participation in TPT was the child’s condition at the time of screening. Parents of healthy children aged under one year appeared to be more likely to decline TPT when offered.

*“Oh*, *the parents usually refuse*. *Because they’re afraid they’re unable to complete the six months of treatment as their children are still young*.*” (Clinic staff*, *nurse)*

*“It’s normal for some parents to refuse to start their child on the treatment because they’re afraid of side effects of the medication if it’s started on a very young child*.*” (Clinic staff*, *nurse)*

On the contrary, parents who agreed to the treatment mostly showed good commitment to completing TPT.

*“If the medication is for prevention*, *I will not refuse*. *We should take this disease seriously*.*” (Guardian of a child eligible for TPT)*

*“I will make sure my kids complete the treatment because I want them to be healthy*.*" (Parent of children eligible for TPT)*

*Knowledge*. Many staff identified a lack of knowledge about TPT and the need for preventive therapy as a factor affecting acceptability of the treatment among parents and guardians.

*“Lack of knowledge is always the first problem for them to accept TPT*.*” (Clinic staff*, *nurse)*

*“Parents who do not know the importance of TPT will indeed feel hesitant to start this child’s treatment*.*” (Clinic staff*, *medical assistant)*

*Misconception*. Misconceptions about the disease and TPT were also implicated as contributing to the acceptability of preventive therapy among guardians.

*“This disease is like a hereditary disease*.*” (Parent of a child eligible for TPT)*

*“There are parents who say that this preventive treatment is not lifelong and it doesn’t protect their child from TB forever*.*” (Clinic staff*, *nurse)*

#### 6. Treatment related factors

Parents and guardians whose children were initiated on TPT indicated that access to the child-friendly form of medication facilitated completion.

*“The medicine is sweet*, *my child likes it*.*” (Parent of a child eligible for TPT)*

*“The medication is in syrup form*. *This helps me to give the medication to my kids easily*.*” (Parent of a child eligible for TPT)*

Nevertheless, the duration of the treatment (6 months) posed a challenge for TPT completion for some parents.

*“The parents usually refuse because they’re afraid of side effects of the medication if they’re unable to complete the 6 months of treatment*.*” (Clinic staff*, *nurse)*

*“If the medicine needs to be consumed over a long period*, *I am not sure whether I can finish it or not*.*” (Parent of a child eligible for TPT)*

Most of the staff cited adverse effects of the medication as a barrier to TPT completion. At the same time, parents highlighted concerns about the potential for adverse effects as a determining factor in their decision to accept TPT.

*“The next reason is that their child cannot tolerate the medication given*. *Some of them are allergic after taking the drug*. *Some of them suffer from diarrhea and vomiting*.*” (Clinic staff*, *nurse)*

*“Not sure yet*. *I will consider it first if there are adverse side effects*. *I will stop taking the medication if any adverse effects occur*. *It’s hard to talk about this for now because I haven’t seen any effects yet*.*” (Parent of a child eligible for TPT)*

## Discussion

This study explored the factors associated with the implementation of TB contact investigation and TPT delivery in selected TB clinics in Kota Kinabalu, Sabah. Based on thematic analyses, identified factors have been grouped into six broad themes: system, clinic, healthcare worker, patient, guardian and treatment related factors.

The COVID-19 pandemic was an unprecedented event that affected the TB control program activities and impacted the whole healthcare system [14]. Scarcity of healthcare resources was already a major challenge in managing TB, and this emerged as an important system-level theme that healthcare workers raised. The burden of COVID-19 disease exacerbated underlying resource limitations and posed a risk of overwhelming the healthcare system. Health inspectors whose role is to perform TB contact tracing were reassigned to COVID-19 contact tracing instead, leading to a decrease in the number of TB contacts identified and screened. In addition, the movement control order (MCO) imposed by the government of Malaysia contributed to the refusal and delay of clinical assessment of people identified as contacts of TB.

The ability to collaborate for extra support in implementing contact investigation with other stakeholders, including NGOs and private practitioners, enables healthcare workers to overcome barriers such as financial disabilities to attend the clinic and lack of diagnostic facilities onsite. Better integration of TB-related services through a public-private mix could significantly improve the delivery of contact investigation and TPT [15]. This finding also highlights the need to collaborate with more NGOs to provide services and health advocacy [16, 17]. The only NGO currently supporting the TB control program in Sabah is the Sabah Tuberculosis Association (SABATA). Nevertheless, relevant stakeholders should take initiatives to improve the equity issue for undocumented migrants who are not eligible for most services provided to Malaysians by SABATA and the State Welfare Department.

Heavy workload among healthcare workers can often result in compromised quality and should be addressed as part of system context reforms to support TB contact management [16]. In our study, this could be explained because the study clinics served a large population catchment area within a locality with a high TB burden. Another reason for the heavy workload was inadequate staffing. Ultimately, both factors affect the quality of care patients receive and put a toll on the staff’s mental health. Staff stated that they lack time to provide adequate information on the value of TPT yet described contacts as ‘uncooperative’ and ‘misinformed’. Evidently, contacts are receiving inadequate information to be able to make informed choices due in part to time pressures relating to under-staffing for the given workload. The unavailability of a clear and easy contact investigation flow and system along with guidelines on TPT implementation was a barrier to contact investigation among healthcare workers. The lack of clarity on some of the provisions of the guidelines meant that staff struggled to effectively implement TPT, which in turn negatively impacted their acceptability of the intervention. This finding resonates with evidence from other studies [18, 19] and echoes the need for better engagement with healthcare workers during the policy development process.

An additional factor affecting the implementation of contact investigation is fear of discrimination [20, 21]. Several TB cases refused to give details of their close contacts due to the fear of being stigmatised by their family members or colleagues, while contacts who were undocumented immigrants experience lingering fear of being caught on their way to the clinic by law enforcement officers, leading to potential detainment and/or deportation back to their country of origin. This, too, was a concern to healthcare workers who cannot ensure the safety and wellbeing of their clients to attend their clinic appointment. The support from family members and the community also plays an important role in the acceptability and accessibility to contact investigation and TPT, which was also highlighted in previous studies [10, 22]. In the district where this study was undertaken, genomic epidemiology reveals that TB transmission occurs across the community, not just confined to households [23]. A key TB control strategy to reduce Malaysian TB rates must comprise effective TB prevention and treatment for everyone regardless of citizenship, without fear of retribution for accessing healthcare.

Client barriers to TB investigation affecting initial access to TB diagnostic services have received considerable attention over the past several years. For example, previous studies have identified that long distances to health centres, direct and opportunity costs associated with seeking TB investigation, and requirements for repeated visits to clinics, delay TB investigation [20]. These factors were well recognized at the system level in our study. Our findings indicate that providers clearly sense that these barriers impact their ability to deliver high quality TB investigation even after patients and contacts arrive at the clinic due to the unavailability of certain diagnostic services onsite.

At the clinics, health staff reported having several duties, and that competing responsibilities often left insufficient time to educate and counsel index patients effectively, much less to collect the information required to initiate contact investigation. Consequently, tasks like TB education and counselling were often viewed as low priority and were neglected. A lack of designated, well-ventilated space in TB clinics also hampered the ability to provide proper education and counselling. In addition, staff sought to minimize the time spent with TB patients to reduce the risk of TB transmission. These findings have also been previously reported in other settings [16, 24].

Although healthcare workers felt they had sufficient knowledge about TB disease, the role of contact investigation, and TPT, they indicated several concerns that challenged their comfort and satisfaction with the intervention. These include lack of cooperation and commitment among multidisciplinary team members, misconception regarding TPT, challenges providing counselling to patients and contacts, and poor communication between relevant stakeholders in contact management. To overcome these barriers, there is a need to focus on creating a series of targeted intervention strategies that have potential to modify healthcare workers’ behaviour by addressing key predisposing, enabling, and reinforcing factors within this setting [24].

Factors relating to the patients and contacts considerably affected healthcare workers’ perceptions on contact investigation and delivery of TPT [24]. Poor cooperation and lack of commitment from contacts to attend clinic appointment for screening were the main concerns cited by healthcare workers. There is a need to empower patients and contacts through TB health promotion to improve their participation. This could be achieved through longer consultation times to provide one-on-one education underpinned by better staffing, public health campaigns appropriately targeted to different cultural groups, peer education groups, or other innovative strategies.

Knowledge about the benefits and effects of TPT was reported to be limited among the parents and guardians. This resulted in misconceptions about TPT among the parents, which led to the refusal to accept preventive treatment for exposed children, even after ‘counselling’. Parents were most reluctant to accept treatment for healthy infants aged less than one year–the age group most at risk after TB exposure, especially in the immigrant population where access to Bacille Calmette-Guérin vaccination at birth is low.

Acceptability of TPT was highly influenced by treatment-related factors, including availability of a child-friendly medication formulation, duration of treatment, and adverse effects, as described in previous studies [25]. In our context, fear of an inability to complete the long duration of treatment and risk of adverse effects on their children influenced parents’ and guardians’ acceptance of TPT. Nevertheless, the syrup form and the sweet taste of the medication contributed to older children’s (more than one year of age) acceptance of TPT.

The specific limitation of this study is social desirability bias. Participants (guardians) may have provided the most palatable responses: for example, citing an inability to get transport to the clinic may be easier than saying they distrusted the clinic. However, efforts were made to ensure that participants were treated respectfully to encourage honest answers, and participants were aware that research staff administering the interviews were independent from the clinic staff.

## Conclusion

This study gives an insight into the complexity of factors affecting TB contact investigation and TPT implementation and the value of qualitative methods in elucidating barriers, challenges, and enablers. Dissemination of the study findings to relevant stakeholders (policymakers and TB program managers) would ensure optimal quality of TB contact investigation and TPT delivery, and by having a high level of commitment among all stakeholders involved in contact management, specific strategies to overcome barriers in implementation can be developed. Additionally, provision of training is needed for healthcare workers to address misconceptions about TB and preventive treatment, safeguard vulnerable clients against the risk of detention and deportation while accessing care, ensure public and private services are provided regardless of migration status and improve processes and resources for contact investigation and preventive treatment.

## Supporting information

S1 File (DOCX)
