# Supplementary material for: Factors affecting implementation of tuberculosis contact investigation and tuberculosis preventive therapy among children in Sabah, East Malaysia: A qualitative study
Source: PLoS One. 2023 May 11;18(5):e0285534. doi: 10.1371/journal.pone.0285534 (PMC10174478; doi:10.1371/journal.pone.0285534)
Supplement: S1 File — (DOCX) [file pone.0285534.s001.docx]

**SEMI-STRUCTURED INTERVIEW GUIDE – HOUSEHOLD CONTACTS**

**QUESTIONS:**

**Can you tell me what you understand about tuberculosis?**

Areas to explore: how effective was the information provided at the time of contact

investigation? What are the main knowledge gaps / misconceptions, which may impact on

acceptance of contact investigation and treatment?

**Do you know how people can get tuberculosis?**

Areas to explore: knowledge about infection control who is at risk

**Do you know why the doctor has recommended tablets for your**

**child/yourself?**

Areas to explore: how effective was the information provided at the time of contact

investigation? Is the person committed to taking, or their child taking, the TB preventive

therapy?

**Did the doctor/nurse explain how long your child/yourself needs to**

**keep taking the medicine?**

Areas to explore: how effective was the information provided at the time of contact

investigation?

**Did the doctor/nurse explain what to do if you get sick while taking the**

**medicine?**

Areas to explore: knowledge about adverse effects / safety

**Do you think your child/yourself will be able to finish the whole course?**

Areas to explore: barriers to treatment completion.

**What makes it easy or hard for you to take the medicine?**

Areas to explore: barriers to and facilitators of adherence in general.

**Is there any extra information you would like?**

Opportunity to address misconceptions or knowledge gaps.

This is the end of the interview. Thank you for your time!

**SEMI-STRUCTURED INTERVIEW GUIDE – HEALTH CARE WORKERS**

**QUESTIONS:**

**Can you tell me what you understand about tuberculosis contact investigation?**

Areas to explore: knowledge of existing guidelines

**Do you think contact investigation is worthwhile?**

Areas to explore: perceived benefit if any of contact investigation; challenges in undertaking

this; challenges engaging with clients; misconceptions about utility.

**How well do you think the clinic does contact investigation currently?**

Areas to explore: barriers and facilitators

**Do you think it needs to be done better? If so, how?**

Areas to explore: addressing staff perceptions; ideas to facilitate improvements; troubleshooting

**Do you have any questions about the Malaysian guidelines or this project?**

Opportunity to address misconceptions or knowledge gaps, motivate good practice.

This is the end of the interview. Thank you for your time
